# Ohio State Veterinary Medical Association

**Published:** 1892-02

**Authors:** Wm. H. Gribble

**Affiliations:** Secretary


*Ohio State Veterinary Medical Association.*—The ninth annual session of the Ohio State Veterinary Medical Association was held in Wells' Post Hall, Columbus, Ohio, January 13, 1892. Meeting called to order at 2 P. M. by President Dr. W. R. Howe, who made a few remarks in reference to the standing of Veterinarians, and asked all to co-operate and try to get legislation providing that all boards of health have at least (if possible) one competent veterinary surgeon in them. Roll-call showed the following members present: Drs. G. W. Butler, E. R. Barnett, S. E. Bretz, T. B. Cotton, W. F. Derr, J. D. Fair, W. H. Gribble, T. B. Hillock, W. R. Howe, Neil Jones, F. J. King, W. A. Labron, M. C. McClain, W. Shaw, W. J. Torrence, J. M. Waddell, W, E. Wight, also Drs. H. M, Ball, L. W. Carl and Dr. Lafferty, and veterinary students Oberholtzer, Ruth, Styer, Mawer, Murray, Harbage, Luck, Early, Smith, from the Ohio-State University.

Minutes of previous meeting read and approved. Election of officers followed, which resulted as follows · President, Dr. W. E. Wight; 1st Vice-President, Dr. W. F. Derr; 2nd Vice-President, Dr, J. D. Fair; 3d Vice-President, Dr. W. J. Torrence; Secretary, Dr. W. H. Gribble; Treasurer, Dr. T. B. Hillock. Dr. H. M. Ball, (American) Columbus, O., and Dr. L. W, Carl, (Ontario) Columbus, O., were proposed and elected to membership, both of whom were introduced, and made a few brief remarks. Dr. G. W. Butler read a memorandum of several interesting cases of death caused by worms, one case in particular in which at P. M. he found fifteen tæniæ and thousands of strongyli. Most members present had had cases which they were unable to diagnose, and which after death proved to be due to pathological changes, etc.., caused by some form of internal parasites. Dr. Shepherd reported a peculiar case of great swelling which he was unable to diagnose, it began in hind limbs, then left those parts and settled at head, causing death, there was no simularity to purpura. Dr. Torrence and others thought it was purpura, because they did not consider petecial spots on the nasal membrane a necessary essential to purpura.

The Chair now appointed a committee to audit the secretary's and treasurer's books and granted a recess while this was being done. On reassembling, committee reported a balance in treasurer's hands of $303.99, and in secretary's hands $31.00. Dr. E. H. Shepherd next read an essay on Equine Dentistry, illustrated by some very fine specimens, especially one, where a seventh molar of right lower jaw had grown until it had worn an opening through the supermaxillary and maxillary sinus into the nasal cavity, The paper was instructive and original and brought out a general discussion. Meeting now adjourned until 7.00 P. M.

Evening session: Meeting called to order at 7.30 P. M. Dr. J. D. Fair read reports of several cases of distokia and inversion of rectum. One case of distokia, the head of fœtus had passed into rectum, projecting from anus, while the front limbs projected through the vulva, an apparently new and undescribed position. The report of the committee on Veterinary Progress was rendered by Dr. Cotton. The committee was not afraid to speak its thoughts on several different points, notably among them being the condemning of the present method of several State Universities, graduating Veterinary surgeons by the tuition of one Veterinary professor. Another being the fact that our Veterinary journals which should do everything in their power to elevate, usually had full-page advertisements of different proprietary medicines and cure-alls. They gave us a history of Veterinary medicine and literature from the time Jacob was successful in getting Laban's cattle, sheep, etc., to conceive and bring forth streaked and striped young ; up to the present date warmly praising the labor of Prof George Fleming, and a warm tribute to Prof. Chauveau for his anatomy, which as yet has no equal. Dr. W. H. Gribble departed from the usual routine of papers and read an essay on the disposal of our dead from an economic and sanitary point of view. He described the different methods in vogue, giving their peculiar and weak points, but warmly advocated cremation, especially as at present conducted, for it left the living none the worse, (in a sanitary sense) for their friends having died. Drs. Newton and Carter asked for withdrawal cards, as they were about to engage in business not strictly professional. The same were granted. It was stated that they proposed to put upon the market a heave cure and a condition powder. It was moved, supported, and unanimously carried that the secretary incorporate in the minutes, and also call the attention of the *American Veterinary Review* and THE JOURNAL OF COMPARATIVE MEDICINE AND VETERINARY ARCHIVES that this Association condemns the action of the above journals in advertising spelterine, creolin, sanitas preparations, etc., deeming it below *the dignity of a professional journal* (*vide* Editorial, 116). Dr. Gribble read obituary of J. B. Hillock, V.S.

Moved, supported and duly carried that the report of committee be accepted. Moved, seconded and unanimously carried that we now adjourn, instructing the secretary to correspond with the secretaries of the State Associations of Michigan and Indiana and try to bring about a joint session sometime during the summer, said time and place to be left to the discretion of the secretary, and advisably to be in a good-sized city during its summer race meeting. Adjourned.

WM. H. GRIBBLE, *Secretary*.

